# An empirical investigation on the impact of attitudes towards organ donation in India

**DOI:** 10.12688/f1000research.131652.1

**Published:** 2023-05-03

**Authors:** Vinod C. Nayak, Smitha Nayak

**Affiliations:** 1 Department of Humanities and Management, Manipal Institute of Technology, Manipal Academy of Higher Education, Manipal, Karnataka, 576104, India; 2 Department of Forensic Medicine and Toxicology, Kasturba Medical College, Manipal Academy of Higher Education, Manipal, Karnataka, 576104, India

**Keywords:** organ donation, attitude, awareness, knowledge, donor card

## Abstract

**Background**: Organ shortage is a global issue and it is imperative to take steps to bridge this gap. In the Indian context, despite its demographic dividend, the rate of organ donation has been abysmally low. This emphasizes a need to demystify the antecedents of organ donation intention among the Indian population.

**Methods: **Using a cross-sectional research design and adopting a post-positivism research philosophy, this study identified 259 respondents by adopting a purposive sampling approach and data on knowledge of organ donation was collected using a structured, pretested questionnaire.

**Results:** *Awareness of organ donation law in India is low on specific issues and respondents from the health science & medicine discipline scored better on knowledge about organ donation. The findings show that most participants had heard about organ donation and had a favourable attitude toward it. *The primary sources of information on organ donation were television and newspapers, and healthcare service providers. A complementary partial median is established (β = .217, t = 5.889, p < .001) which implies that willingness to discuss with family significantly mediates the association between attitude towards organ & tissue donation and willingness to sign the donor card.

**Conclusion**: This study revealed that there is a general awareness of organ and tissue donation among the Indian population, but they lack clarity on certain specific issues. Mass media has to be effectively used to build awareness campaigns revolving around enhancing knowledge on a specific issue and building acceptance of the concept of organ and tissue donation.

## Introduction

Organ donation is a significant milestone in the process of evolution of modern medicine. The act of donating an organ or a portion of an organ for transplantation to another person is known as organ donation. The Transplantation of Human Organs Act (Section 2), 1994, defines “transplantation as the grafting of any human organ from any living person or deceased person to some other living person for therapeutic purposes”. This is the only way to prolong life and enhance the quality of life for people with terminal organ failures ( Bedi *et al.*, 2015). When Joseph E Murray and his colleagues performed the very first successful kidney transplant in Boston in 1954, the concept of organ donation gained traction ( Watts, 2011). In the seven decades since organ transplantation has emerged to be a successful practice worldwide with kidneys, liver, and heart being the most transplanted solid organs ( WHO, 2020) and musculoskeletal grafts & cornea being the most transplanted tissues. The International report on Organ Donation and Transplantation Activities ( WHO, 2022) reported 129681 solid organ transplants globally in 2020. Despite this number of reported donations, there is still a supply-demand imbalance in the organ and tissue donation (OTD) space. The report further cites 17 deaths occurring every day due to the non-availability of organ donors and the existing donations are only catering to less than 10 percent of the global need. There is a significant difference between nations in the availability of appropriate transplantation procedures as well as in the level of safety, effectiveness, and quality of human cells and, OTD & transplantation. These systemic factors contribute to broadening the need-availability gap.

## Literature review

In the Indian context, despite its demographic dividend, the rate of organ donation has been abysmally low. There is a significant mismatch between the demand and supply status in India. Despite the fact that approximately 180,000 people are estimated to suffer from renal failure each year, with only 6,000 kidney transplants are performed ( Chang *et al*., 2020). A timely liver transplant could save approximately 15% of the two lakh individuals who succumb in India every year from liver cancer or liver failure. As a result, India requires between 25,000 to 30,000 liver transplants per year, but only about 1,500 are carried out. Similarly, over 50,000 people die from heart failure each year in India, but only 10% to 15% of heart transplants are performed ( Tamuli *et al.,* 2019). Despite a 100,000 need, only approximately 25,000 Cornea transplants are performed each year ( Mohan *et al*., 2020). These figures are not encouraging, regardless of the efforts of the government and voluntary organizations. The government has attempted to bring in a systemic change to ensure a transparent system of OTD and to stop human organ trafficking. The Government legalized organ donation under the “Transplantation of Human Organs Act” ( THOA, 1994), and the Act has undergone various amendments since then The National Organ Transplant Program was initiated in the 12 ^th^ five-year plan of the Government of India to promote organ transplantation ( THOA, 1994). The National Organ and Tissue Transplantation Organization (NOTTO) has been established as a nodal agency for this program, and it serves as a national-level registry for organ donation and transplantation.

Organ shortage is a global issue, and it is imperative to take steps to bridge this gap. The Government and Non-Governmental organizations have in tandem taken various steps to encourage people to register for organ donation. Several initiatives that spread fundamental knowledge, and enhance familiarity with and develop positive attitudes toward organ donation have been adopted. For instance, legalising organ donation and organ donation awareness campaigns on various media platforms are examples of initiatives that have been implemented by private and public organizations ( Meena *et al*., 2023).

Intention to donate organs is shaped by an individual's knowledge of organ donation, attitude, and behaviour, and is further influenced by cultural and religious orientations ( Chung *et al*., 2008; Rithalia *et al*., 2009; Mekahli *et al*., 2009; Ramadurg & Gupta, 2014; Manish *et al*., 2015; Vijayalakshmi *et al.*, 2016, Vincent *et al.*, 2022). Age, gender and socio-economic status, and education have been reported to have a significant influence on attitudes toward organ donation ( Bilgel *et al.*, 2004; Spigner *et al.*, 2002; Saleem *et al.*, 2009; Balwani *et al.*, 2015). A study undertaken by Gorczyca and Hartman (2017) emphasized the importance of including millennials in a larger conversation about organ donation and transplantation as they could contribute to future campaign tactics at local and national governmental levels. As India has a 34.8 percent representation of millennials in the population, or people aged 17 to 34 (according to the data furnished by the Social Statistics Division of the Central Statistics Office, Ministry of Statistics and Programme Implementation, Government of India, as of 2011), they could contribute to accelerating reach of the campaigns. In a similar study, to this evidence, undertaken by the United States Department of Health and Human Services (2013), it was identified that people above the age of 65 were less likely to sign up for organ donation compared to those of the age group 18-34 years. Loughery *et al*. (2018) have also cited the age and education status of the donor as significant barriers to organ donation. Additionally, a study undertaken by Alex *et al*. (2017) provided evidence of an inadequate level of knowledge on organ donation among medical students served as a barrier to OTD. Hence it is imperative to assess the current knowledge level, attitude, and willingness to register for organ donation among various age groups and whether it varies across the population with the discipline of education.

**Figure 1.**
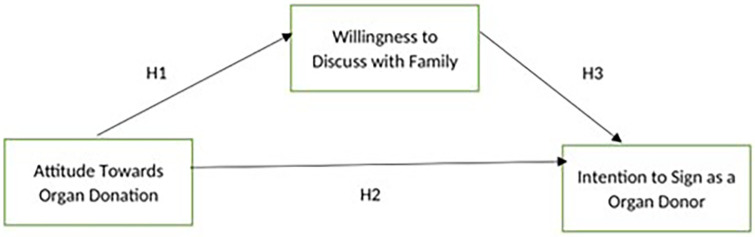
Conceptual Framework.

There exists sufficient research evidence to conclude a positive significant association between willingness to discuss OTD with family and attitudes toward OTD ( Knox *et al.*, 2017; Kopfman, 2002; Thompson *et al*., 2004; Wu & Tang, 2009). Although this association has been established, the details of the discussion are still an enigma. Most of the research evidence indicates a similar influence on the intention of organ donation, although there is a lack of empirical evidence proving this hypothesis. Knowledge of organ donation also has a positive association with discussion intention ( Guadagnoli *et al*., 1999). Among gender, women were more likely to discuss organ donation intention than men ( Thompson *et al*., 2004). Even though there is a direct effect of willingness to discuss with family on intention to enrol as an organ donor, the mediating role of this construct has not been explored. This study will also explore the mediating role of willingness to discuss organ donation between the attitude toward OTD and the intention to enrol as an organ donor. The conceptual framework that has been proposed is shown below ( Figure 1).

## Methods

### Ethics

This study was reviewed and approved by the Scientific Committee Manipal Academy of Higher Education, Manipal, India on 16 ^th^ March 2022. While administering the questionnaire, written informed consent was taken from the participants. Participants were ensured that the collected data would be used for research purposes and that the data would be sufficiently de-identified.

### Study design

This proposed research endeavour is cross-sectional by research design and is quantitative by nature. The “post-positivism” research philosophy that is being considered in this study concerns the development of empirical methods for comprehending and investigating human behaviour. The conceptual framework for this study proposes a relationship between the independent, dependent, and mediating factors. Demographic factor like education is considered moderating variables.

### Study setting

As per the report submitted by the Health and Family Welfare, Ministry of Karnataka, Karnataka state has been one of the forerunners in terms of organ donation in India ( Kute *et al*., 2020). As per the current statistics, 143 organ donations have been recorded in 2022 which has given a fresh lease of life to 397 people. Hence, this study was undertaken in Karnataka state. In Karnataka, Dakshina Kannada” and Udupi Districts were chosen by a simple random sampling technique. The target population for this study was the general population residing in the above districts who were free of organ failure and who knew English. No other inclusion criteria were adopted. A purposive sampling technique was adopted to identify participants at four shopping malls; two located in each district. Participants were approached at the malls and after applying the inclusion criteria, were briefed on the objective of the research. On obtaining consent, the participants were requested to fill the questionnaire. Data collection was undertaken from November 2022 to January 2023.

### Sampling

This investigation used a purposive sampling approach (non-probability by nature), which was adopted due to the absence of a sampling frame. Data was collected from 259 respondents. The study comprises of respondents from three age groups (18-26, 27-42, and 42 and above). Respondents who know English were requested to fill the questionnaire.

The sample size was determined by multiplying the number of rating scale elements by ten [15] i.e. 15*10 = 150. Considering 10% of the unanswered sample (i.e.15) gives rise to 165 (150 + 15 = 165). Finally, 259 participant data were collected.

### Data gathering

Data was collected using a structured questionnaire. A copy of the questionnaire is also placed the *Extended data* (Nayak & Nayak, 2023). The questionnaire was divided into five parts. Part 1 captured the demographic details of the participants, like age, income, education, and gender. In addition, two questions on participants' awareness of OTD and sources of information on OTD were also incorporated. Part 2 assessed the knowledge of participants on OTD Jacob *et al*. (2008). This section included eight items with true or false response options, which were further subdivided into four sub-scales: general donation-related statistics (two items), understanding of what signing a donor card entails (three items), medical fitness for donation (two items), and knowledge of religious institutions' approach to donation (one item). The maximum score in this component was 8, with one mark for each correct response. The third part of the questionnaire consisted of seven items to measure attitudes toward OTD ( Morgan & Miller, 2001). The next section of the questionnaire inquired about the participant's willingness to talk about OTD with family and friends ( Morgan & Miller, 2002). The final part of the questionnaire captured the participant's willingness to sign the organ donor card adopted from Horton and Horton (1991). All responses were coded using a 5-point Likert scale, with 1 representing strong disagreement and 5 representing strong agreement.

### Data collection

Prior to the final data collection, the research instrument was subject to a pilot test to assess construct validity. Data were collected from 25 respondents through a purposive sampling technique. Cronbach Alpha was estimated to establish construct validity using IBM SPSS Statistics 27 (RRID: SCR_016479; Armonk, NY: IBM Corp). Cronbach alpha of the endogenous construct (willingness to sign the organ donor card = 0.875) and exogenous constructs (attitudes towards OTD=0.724& willingness to discuss OTD=0.867) were well above 0.7, indicating acceptable levels of internal consistency. Loadings of all factors and communalities were significantly greater than 0.5, and the "Kaiser-Meyer-Olkin and Bartletts Test" was significant (>0.8).

### Data analysis

The negatively worded items were reverse coded and considered for final data analysis. IBM SPSS Statistics 27 (RRID: SCR_016479; Armonk, NY: IBM Corp) RRID: SCR_016479; Armonk, NY: IBM Corp) was used to undertake a descriptive analysis of the demographic variables and is presented in the results section. To test the hypotheses and perform the mediation analysis, the statistical package SmartPLS4 (RRID: SCR 022040) was used. PLS-SEM is becoming more popular as a statistical package due to its versatility and dependability in analyzing composite and empirical studies. Structural Equation Modelling performed in this study can also be undertaken by using jamovi (RRID:SCR_016142), which is a free source software.

## Results

The demographic characteristics of the participants were analysed using IBM SPSS Statistics 27 software ( Table 1). For the complete dataset, see *Underlying data* (Nayak & Nayak, 2023). Of the 259 respondents, 173 (67%) were female and 171 were between 18 to 26 years of age. 113 respondents had enrolled or completed their Bachelor's education in the healthcare domain like medicine, life science, nursing, health science, and allied health ( Table 1). All the participants reported that they are aware of organ and tissue donation but only 125 (41%) stated that they were aware of OTD ( Table 2). A “chi-square test of independence” displayed that there was a significant association between education and awareness of the OTD act in India Χ2 (1, N = 259) = 6.980, p = 0.06).

**Table 1.** Demographic characteristics of the participants (N = 259).

Demographics	Components	N	%
Gender	Female	173	67
	Male	86	33
Age	18-26	171	66
	27-42	49	19
	42 & above	39	15
Education	Healthcare domain	113	44
	Non-Healthcare domain	146	56
Income	Below Rs 2,00,000	89	34
	Rs 2 to 5,00,000	56	22
	Rs 5 to 10,00,000	50	19
	Above Rs 10,00,000	64	25

**Table 2.** Awareness of OTD (N = 259).

Age	Aware of OTD	Aware of organ donation law	
	Yes	Yes	No
18-26	171	73	98
27-42	49	22	27
42 & above	39	30	9
Total	259	125 (41%)	134 (51%)

Participants' knowledge of OTD was assessed by posing eight questions ( Table 3). A majority (96.5%) of the participants were aware of the demand for transplants exceeding availability. They were also aware of the fact that many people were losing their lives due to the non-availability of organs to be transplanted (91.15%). Three questions were posed to the respondents to assess their knowledge of the implications of signing a donor card. The majority (95.8%) were aware they could specify the organs and tissues they want to donate on the donor card but only 33% of them knew that they could change their mind after signing the donor card. Two-thirds of the participants thought that the next of kin (family) must permit donation to occur even if the donor had signed the donor card. Participants were also assessed on their knowledge of how medical viability for donation is determined. More than half of the participants (51.7%) were not aware that the age or medical condition of the donor is important antecedent of becoming an organ donor. Whereas 82% of the participants knew that signing up for organ donation would not change the line of treatment they would receive in the future. Around 68% of the respondents believed that religious people would not oppose OTD. However, standard deviation and mean of the knowledge score was estimated to be 1.116 and 5.25.

**Table 3.** Assessment of participants' knowledge of OTD.

Item	True	False
Donation-Related (General Awareness)		
K1: For most organs, the demand for transplants is higher than the availability	250 (96.5)	9 (3.5)
K2: People on the waiting list for a transplant die every day because not enough organs are available	236 (91.1)	23 (8.9)
Knowledge of the implications of signing a donor card		
K3: A person can specify on a donor card what organs and tissues they want to donate	248 (95.8)	11(4.2)
K4:Next of kin (family) must permit donation to occur even if I sign a donor card	165 (61.3)	94 (38.7)
K5: Once a person has signed a donor card they cannot change their mind about organ donation	173 (66.8)	86 (33.2)
Knowledge of how medical viability for donation is determined		
K6. Anyone can decide to be a donor regardless of their age or medical condition	125 (48.3)	134 (51.7)
K7. Signing a donor card will not change the medical treatment I receive to save my life at the hospital	230 (88.8)	29 (11.2)
Knowledge Religious implications to donation		
K8. Most religious people oppose organ and tissue donation	82 (31.7)	117 (68.3)
Mean (SD) of overall knowledge score	5.25 (1.116)	

### Reliability and validity analysis

**Figure 2.**
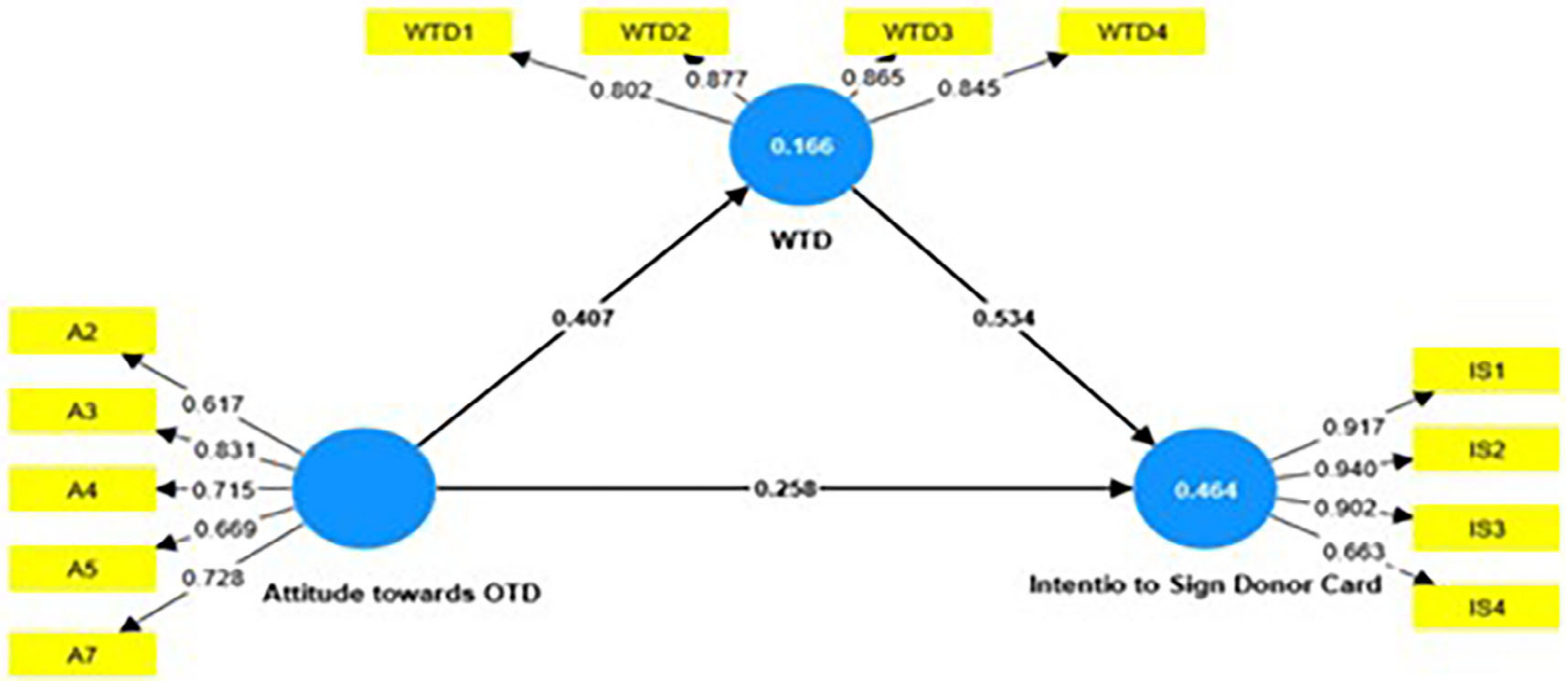
Structural Model Evaluation.

The measurement model was evaluated to determine the constructs' reliability and validity. ( Table 4) and the Structural Model evaluation is presented in Figure 2. First, the factor loadings of all items in the model were checked to ensure that they were greater than the minimum acceptable value of 0.5 ( Hair *et al*., 2017). Vinzi *et al*. (2010) opine a minimum acceptable factor loading of 0.7 but in social science studies, researchers frequently obtain lower outer loadings. Instead of automatically eliminating the indicators, it would be better to assess the impact of their elimination on the improvement in the reliability and validity of the constructs. Hair *et al.* (2017) recommend the elimination of the indicators with factor loading in the range of 0.4 to 0.7 only if it increases the value of composite reliability (Average Variance Extracted) beyond the threshold value. In our research endeavour, the removal of item (A1, loading = 0.504 & A6, loading =0.431) had a significant influence on composite reliability and hence was eliminated.

**Table 4.** Evaluation of the Measurement Model.

		Outter Loadings	Cronbach's alpha	Composite reliability (rho_a)	Composite reliability (rho_c)	Average variance extracted (AVE)	Outter Weights	VIF
Attitude towards OTD	A2	0.617 ***	0.759	0.772	0.839	0.512	0.25	1.283
	A3	0.831 ***					0.314	1.901
	A4	0.715 ***					0.296	1.5
	A5	0.669 ***					0.207	1.43
	A7	0.728 ***					0.322	1.37
Intention to sign	IS1	0.917 ***	0.88	0.906	0.92	0.745	0.297	4.417
	IS2	0.94 ***					0.323	4.238
	IS3	0.902 ***					0.31	3.052
	IS4	0.663 ***					0.217	1.365
WTD with family	WTD1	0.802 ***	0.872	0.887	0.911	0.719	0.802	1.519
	WTD2	0.877 ***					0.877	3.127
	WTD3	0.865 ***					0.865	3.312
	WTD4	0.845 ***					0.845	2.325

*** p < 0.01.

** p < 0.1.

Cronbach's alpha, rho a, and composite reliability were used to assess reliability; statistical values were higher than the recommended threshold value of 0.7 ( Wasko & Faraj, 2005). The value of rho_a was found to be in between composite reliability (Sarstedt *et al*., 2017) and Cronbach’s alpha, and was also above 0.7 indicating excellent reliability ( Henseler *et al*., 2016). “Average Variance Extracted” (AVE) was higher than 0.5 hence establishing convergent validity. Fornell & Larcker method was employed to establish discriminant validity ( Table 5).

**Table 5.** Discriminant validity.

Fornell Larcker			
	Attitude towards OTD	Intention to sign	WTD with family
Attitude towards OTD	0.716		
Intention to Sign	0.475	0.863	
WTD with family	0.407	0.639	0.848

Note: The diagonal italic is the square root of AVE. Below the diagonal elements are the correlations between the construct’s values.

### Structural model

The structural model reflects the hypothesised path of the research framework. R ^2^, Q ( Ahlawat *et al.*, 2013), and the significance of the paths are used to evaluate the structural model. The R2 value, which indicates the strength of each structural path in the model, can be used to assess a model's goodness. Falk and Miller claim (1992), the R ^2^ value should be equal to or above 0.1. This establishes the predictive capacity of the model. Q ^2^ value is used to establish the predictive relevance of the endogenous construct. Q ^2^ value above 0 indicates the predictive relevance of the construct. In the current study, both R ^2^ and Q ^2^ values are above this threshold value ( Table 6) hence indicating the goodness of the model. Furthermore, the “model fit” was evaluated using the Standard Root Mean Square Residual (SRMR). The value of the SRMR residual was 0.084 which was well within an acceptable range ( Hair *et al.*, 2017).

**Table 6.** Testing Direct Relationships.

	Path coefficient	Standard deviation (STDEV)	t value (bootstrap)	P values	BI
H1: Attitude towards OTD -> Intention to Sign Donor Card	0.258 ***	0.049	5.220	0.000	(0.159, 0.351)
H2: Attitude towards OTD -> WTD	0.407 ***	0.062	6.535	0.000	(0.237, 0.518)
H3: WTD -> Intention to Sign Donor Card	0.534 ***	0.054	9.851	0.000	0.422, 0.636)
R ^2^ Willingness to Discuss = 0.464	Q ^2^ Willingness to Discuss = 0.152				
R ^2^ Intention to Sign Donor Card = 1.666	Q ^2^ Intention to Sign Donor Card = 0.215				

Abbreviation: BI, bias-corrected.

*** p < 0.01.

** p < 0.1.

In addition, the hypothesis was tested to explore the significance of the relationship. H1 evaluates if the attitude towards OTD has a significant impact on the intention to sign the donor card. Results revealed that attitude towards OTD has a significant impact on intention to sign the donor card (β = .258, t = 5.220, p < .001). Hence H1 was supported. H2 proposed an association between attitude towards OTD and willingness to discuss with family and was also supported (β = .407, t = 6.535, p < .001). Results also revealed that willingness to discuss with family had a significant positive association with intention to sign the donor card (β = .534, t = 9.851, p < .001). Table 6 shows the 95% confidence interval generated by the study of 5000 resamples. A confidence interval that is not zero indicates that there is a significant relationship.

### Mediation analysis

Mediation analysis was performed to check if the willingness to discuss with family mediates the association between attitude towards OTD and willingness to sign the donor card. A complementary partial median is established (H4: (β = .217, t = 5.889, p < .001) which implies that willingness to discuss with family significantly mediates the association between attitude towards OTD and willingness to sign the donor card.

To explore if the attitude towards OTD varied across participants from the health science domain and engineering, management, and humanities, the Mann-Whitney U Test was utilized. The test revealed a significant difference in OTD among participants from the health science domain (Media = 30, n = 113) and engineering, management, and humanities (Media = 29, n = 146), U = 6838, z = 2.367, p = 0.018, r = 0.14). Hence, we can conclude that the discipline of education has a significant influence on attitude towards OTD. Even though education is determined to increase knowledge on OTD, it is suggested by Meier *et al*. (2000) that most donors do not explore organ donation before pledging to donate the organ.

### Importance Performance Matrix Analysis

**Figure 3.**
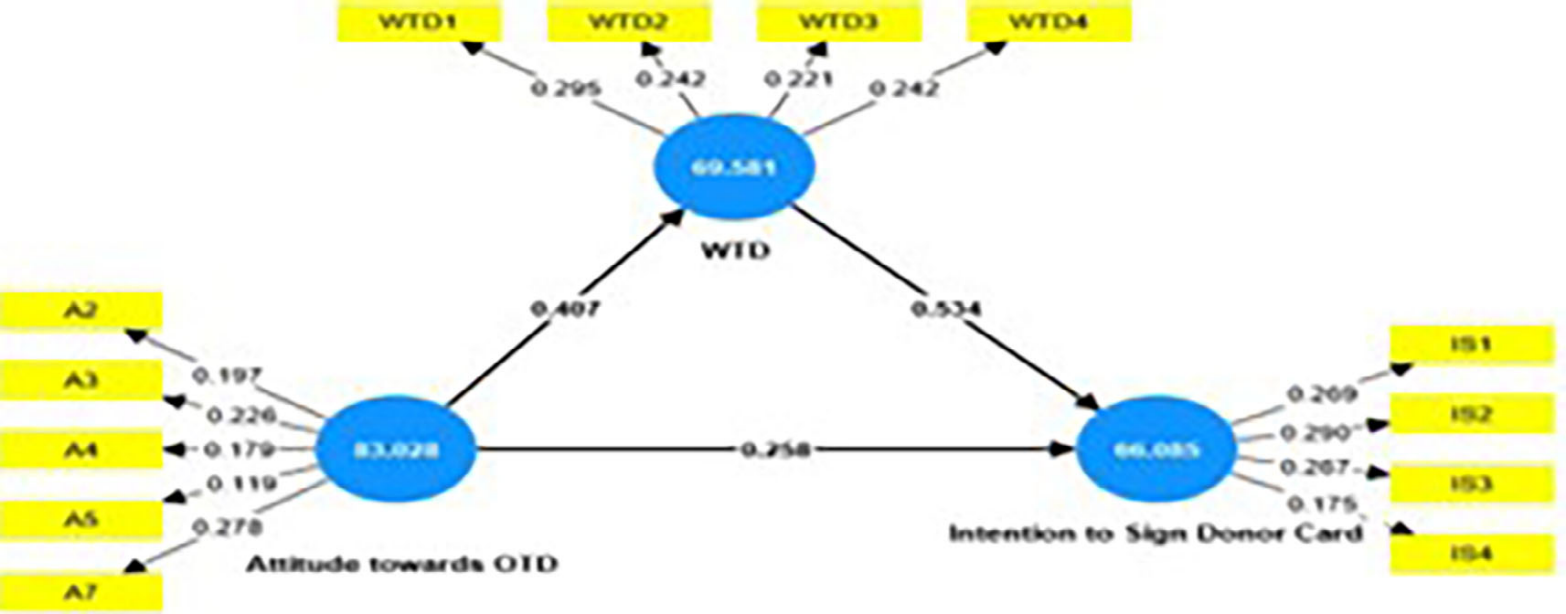
IPMA Analysis.

**Figure 4.**
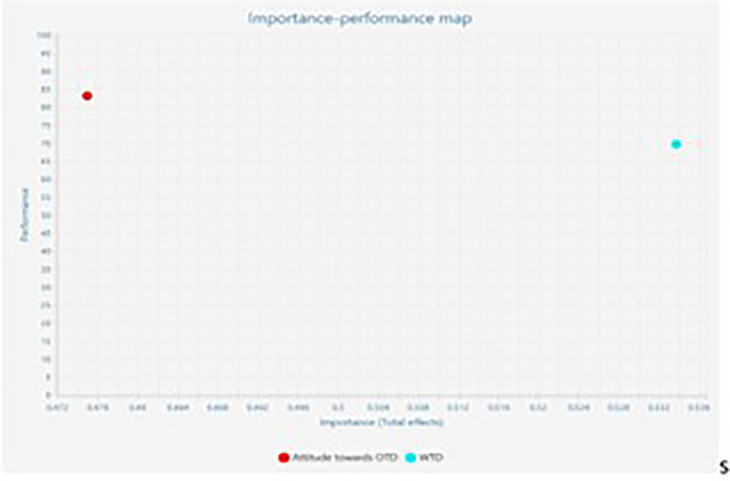
Importance Performance Map.

The Important-Performance Matrix Analysis (IPMA) elucidates the relative importance and performance of exogenous (attitude towards OTD & willingness to discuss with family) and endogenous constructs (intention to sign the OTD card) in relation to one another. Total effects and index values represent their significance and performance. Importance reveals the overall effect on the endogenous variable. The performance demonstrates the potential of latent variable scores. The X and Y axes are used to quantify importance and performance. The total effect is represented by the X-axis, while performance is represented by the Y-axis. A construct performs better when it has a higher mean value. (Hair *et al*., 2016; Hock *et al*., 2010; Rigdon *et al*., 2011; Schloderer *et al.*, 2014; Volckner *et al*., 2010) ( Figure 3 and Table 7). The results of IPMA analysis reveal that willingness to discuss with family displays a high performance of 69.581 and a high total effect of 0.534 in comparison with the other exogenous (attitude towards OTD) displaying a performance of 83.028 and a total effect of 0.475. Hence a unit of increase in performance of attitude towards OTD enhances intention to sign donor card from 66.087 to 66.562. Similarly, an increase in one unit of construct, willingness to discuss with family would increase the performance of construct intention to sign OTD card by 0.534 points to 66.621 points. Figure 4 represents the IPMA map.

**Table 7.** IPMA: Outcome variable – Intention to sign OTD card.

Exogenous Constructs	Total Effect	Performance
Attitude towards OTD	0.475	83.028
WTD	0.534	69.581

## Discussion

This study intended to explore the knowledge of OTD across age groups in the Indian population. The goal was to assess the general public's knowledge and attitude toward organ donation. The findings show that most participants had heard about organ donation and had a favourable attitude toward it. These research findings are similar to results obtained by previous studies by Vijayalakshmi *et al.* (2016), Mithra *et al.* (2013), Sarveswaran *et al*. (2018). Results show that people are aware of OTD but only 41% were aware of the organ donation law in India. Awareness of THOA in 1994 was astonishingly low. The knowledge score was moderate across the population of the study.

In line with previous studies, we observed that the primary sources of information on organ donation were television and newspapers, healthcare service providers, and followed by social media and the internet. In line with the previous studies, healthcare service providers played an integral role in creating awareness of OTD ( Vijayalakshmi *et al*., 2016; Narendran *et al.*, 2022). They could be the primary motivators in raising awareness among their family, friends, relatives, and neighbours. A recent 5-year retrospective case record analysis revealed that only 10 (5%) of 205 patients diagnosed as brain dead had their organs donated ( Sawhney *et al.*, 2013). This study again highlights the lack of awareness of the concept of organ donation and the need for the relevant stakeholders to collectively work together to strengthen awareness of OTD. As a result, both governmental and non-governmental organizations should play an active role in raising public awareness about brain death. THOA was enacted in 1995, but only 42% of participants were aware of organ donation legislation. These findings were comparable to previous studies that found 5.7% ( Vijayalakshmi *et al*., 2016) & 13.9% ( Pouraghaei *et al*., 2015). The stakeholders could adopt television and digital campaigns to enhance awareness of OTD, the process involved, and THOA. According to the Global Overview report 2022, 35% of the Indian population resides in the urban landscapes of the country and most of this population has access to television, the internet, and social media ( Digital 2022 Global Overview Report, 2022). Hence, emphasis on television and digital campaigns could contribute strongly to creating awareness and registering for OTD as most of the participants (96%) are aware that people are dying due to lack of availability of organs. In general, the donor will be given the organ donation card after signing it, and if the donor changes their mind about donating organs could tear up the organ donation card ( Gungormüs & Dayapoglu, 2014). The majority of our study's participants (66%) believed that once they signed the organ donation card, they couldn't change their minds. As a result, these issues must be addressed when creating public awareness programs. Issues concerning the signing of organ donation cards should be clarified by the government and non-governmental organizations. 68% of all those surveyed believed that various religions oppose OTD. These findings were consistent with the documented literature, which indicated that religious beliefs were the most significant barrier to organ donation ( Patthi *et al.,* 2015; Vijayalakshmi *et al*., 2016). In India, the family traditionally looks after its members, even when they are sick. As a result, the consent of the next of kin is required for organ donation from a deceased donor ( Ahlawat *et al*., 2013). Moreover, 61% of respondents agreed that knowing their family's wishes after their death was important. As a result, family members must have a positive attitude toward organ donation. Around one-third of the respondents thought that various religions opposed OTD. These findings echo the findings of the research endeavours of Vijayalakshmi *et al*. (2016) and El-Menyar *et al*. (2020). Religious prohibitions were reported as a cause of not donating an organ by Hameed *et al*. (2016).

In line with the previous research evidence ( Vijayalakshmi *et al*., 2016; Pouraghaei *et al*., 2015; Narendran *et al*., 2022) it is proved that attitude toward organ donation is significantly associated to becoming an organ donor (Melissa *et al.,* 2013; Teoh *et al*., 2020). In research studies undertaken in India, all have reported positive attitudes toward OTD and a considerable amount of awareness of OTD. The challenge encountered in developing regions like India is that despite the population being considerably aware of OTD, very few registered for organ donation. Having a positive attitude toward OTD is a significant antecedent to registering for OTD. The research undertaken by Topic *et al*. (2006) and Sengul and Sahin (2022) present evidence for the fact that the nature of education was significantly associated with attitude towards OTD. It was observed that health science professionals had a more positive attitude towards OTD in comparison to their colleagues from other professional disciplines. Our study echoes this evidence as it is observed that respondents from a medical and health science domain had a more positive attitude towards OTD. Hence there is a need to create awareness and build a positive attitude toward OTD among the non-health/medical science population as attitude defines the intention to be an organ donor.

Willingness to discuss with family is reported to have a positive mediation between attitude towards OTD and intention to be an organ donor. Hence there is a need for government and non-governmental stakeholders to normalize the discussion on OTD in society. There appears to be a lack of consideration for the topic which creates a communication barrier ( Feeley & Servoss, 2005). An OTD-friendly environment and family can increase the willingness to donate organs. Seeing family approval and willingness may help individuals make positive decisions about the subject ( Sengul & Sahin, 2022) As a consequence, it is essential to explain the issue to all family members through community educational activities using plans and brochures for all family members. It may be beneficial to plan mass communication campaigns to strengthen acceptance of the topic among the population at large.

## Conclusion

This study provides several insights into the OTD scenario in India. This study revealed that there is a general awareness of OTD among the Indian population but they lack clarity on certain specific issues. The majority of participants were unaware of the legislation or the donation process & clarity on the role of family when the donor had signed the donor card was ambiguous. It was established that the willingness of the donor to discuss with family played an instrumental role in strengthening the intention to donate an organ. Attitudes toward organ donation was significantly different among people with health science or medical backgrounds and from engineering, management, and humanities domains. This situation highlights the need for governmental and non-governmental institutions to take up the mandate of creating awareness that could lead to bridging the demand-supply gap of organs in India.

The cross-sectional research design serves as a limitation of this research endeavour. Further studies could adopt qualitative or experimental approaches to explore the rationale of OTD. Future studies could investigate if the knowledge, attitude towards OTD, and organ donation intention varied across Generation Z (born between 1996-2000) and Millennials (born between 1981 to 1995) ( Thangavel *et al*., 2021). This will enable stakeholders to design appropriate campaigns and adopt the most appropriate media to create awareness of OTD thereby bridging the demand-supply gap.

## Data Availability

Figshare: Dataset, https://doi.org/10.6084/m9.figshare.22099580 (Nayak & Nayak, 2023). This project contains the following underlying data: • Organ Donation Raw Data.xls Figshare: Dataset, https://doi.org/10.6084/m9.figshare.22099580 (Nayak & Nayak, 2023). This project contains the following extended data: • Questionnaire with codes.docx Data are available under the terms of the Creative Commons Attribution 4.0 International license (CC-BY 4.0).

## References

[ref2] Ahlawat R Kumar V Gupta AK : Attitude and knowledge of healthcare workers in critical areas towards deceased organ donation in a public sector hospital in India. *The National Med. J. India.* 2013;26(6):322–326. 25073987

[ref1] Alex P Kiran KG Baisil S : Knowledge and attitude regarding organ donation and transplantation among medical students of a medical college in South India. *Int. J. Community Med. Public Health.* 2017;4(9):3449–3454. 10.18203/2394-6040.ijcmph20173860

[ref26] Balwani MR Gumber MR Shah PR : Attitude and awareness towards organ donation in western India. *Ren. Fail.* 2015;37(4):582–588. 10.3109/0886022X.2015.1007820 25656835

[ref3] Bedi KK Hakeem AR Dave R : Survey of the knowledge, perception, and attitude of medical students at the University of Leeds toward organ donation and transplantation. *Transplantation proceedings.* Elsevier;2015, March; (Vol.47(No.2): pp.247–260). 25769557 10.1016/j.transproceed.2014.11.033

[ref4] Bilgel H Sadikoglu G Goktas O : A survey of the public attitudes towards organ donation in a Turkish community and of the changes that have taken place in the last 12 years. *Transpl. Int.* 2004;17(3):126–130. 10.1111/j.1432-2277.2004.tb00416.x 14745490

[ref5] Chang JH Diop M Burgos YL : Telehealth in the outpatient management of kidney transplant recipients during the COVID-19 pandemic in New York. *Clin. Transpl.* 2020;34(12):e14097. 10.1111/ctr.14097 32940919

[ref6] Chung CK Ng CW Li JY : Attitudes, knowledge, and actions with regard to organ donation among Hong Kong medical students. *Hong Kong Med. J.* 2008;14(4):278–285. 18685160

[ref7] Digital 2022 Global Overview Report: We Are Social and Hootsuite. 2022. (accessed on February 2,2023). Reference Source

[ref8] Esposito Vinzi V Trinchera L Amato S : PLS Path Modeling: From Foundations to Recent Developments and Open Issues for Model Assessment and Improvement. Vinzi VE Chin WW Henseler J , editors. *Handbook of Partial Least Squares: Concepts, Methods and Applications.* Berlin, Germany: Springer Berlin Heidelberg;2010; (47–82).

[ref9] El-Menyar A Al-Thani H Mehta T : Beliefs and Intention to Organ Donation: A Household Survey. *Int. J. Appl. Basic Med. Res.* 2020;10(2):122–127. 10.4103/ijabmr.IJABMR_108_19 32566529 PMC7289197

[ref11] Feeley TH Servoss TJ : Examining college students' intentions to become organ donors. *J. Health Commun.* 2005;10(3):237–249. Global overview report. 10.1080/10810730590934262 Reference Source 16036731

[ref12] Falk RF Miller NB : *A primer for soft modeling.* University of Akron Press;1992.

[ref13] Guadagnoli E Christiansen CL DeJong W : The publics willingness to discuss their preference for organ donation withfamily members. *Clin. Transpl.* 1999;13(4):342–348. (1) (PDF) A Systematic Literature Review and Research Agenda for Organ Donation Decision Communication. [accessed Dec 22 2022]. 10.1034/j.1399-0012.1999.130411.x Reference Source 10485377

[ref14] Gorczyca M Hartman RL : The new face of philanthropy: The role of intrinsic motivation in millennials’ attitudes and intent to donate to charitable organizations. *J. Nonprofit Publ. Sect. Market.* 2017;29(4):415–433. 10.1080/10495142.2017.1326349

[ref15] Gungormüs Z Dayapoglu N : The knowledge, attitude and behaviour of individuals regarding organ donations. *TAF Prev. Med. Bull.* 2014;13:133–140. 10.5455/pmb.1-1358319696

[ref16] Hair JF Jr Hult GT Ringle CM : *A primer on partial least squares structural equation modeling (PLS-SEM).* 2nd ed. Sage publications;2017.

[ref17] Hamed H Awad ME Youssef KN : Knowledge and attitudes about organ donation among medical students in Egypt: A questionnaire. *J Transplant Technol Res.* 2016;6(1):1–4.

[ref10] Henseler J Hubona G Ray PA : Using PLS path modeling in new technology research: updated guidelines. *Ind. Manag. Data Syst.* 2016;116:2–20. 10.1108/IMDS-09-2015-0382

[ref18] Hock C Ringle CM Sarstedt M : Management of multi-purpose stadiums: Importance and performance measurement of service interfaces. *Int. J. Serv. Technol. Manag.* 2010 Jan 1;14(2-3):188–207.

[ref19] Horton R Horton P : A model of willingness to become a potential organ donor. *Soc. Sci. Med.* 1991;33:1037–1051. 10.1016/0277-9536(91)90009-2 1771431

[ref21] Kopfman JE Jansky SA : The influence of family discussionon individual intent to become a potential organ donor: usingTheory of Reasoned Action’s subjective norm to changeintent. Paper presented at: Annual Convention of the NationalCommunication Association, New Orleans, La. November 2002.

[ref22] Knox K Parkinson J Pang B : A systematic literature review and research agenda for organ donation decision communication. *Prog. Transplant.* 2017;27(3):309–320. 10.1177/1526924817715459 29187065

[ref24] Kute V Ramesh V Shroff S : Deceased-donor organ transplantation in India: current status, challenges, and solutions. *Exp. Clin. Transplant.* 2020;18(Suppl 2):31–42. 10.6002/ect.rlgnsymp2020.L6 32758118

[ref25] Loughery C Zhang N Smith AH : Organ donation attitudes and practices among older adults participating in evidence-based health programs. *Arch Transplant.* 2018;2:1.

[ref27] Mekahli D Liutkus A Fargue S : Survey of first-year medical students to assess their knowledge and attitudes toward organ transplantation and donation. *Transplantation proceedings.* Elsevier;2009, March; (Vol.41(No.2): pp.634–638). 10.1016/j.transproceed.2008.12.011 19328942

[ref28] Meena P Kute VB Bhargava V : Social media and organ donation: Pros and cons. *Indian J. Nephrol.* 2023;33(1):4–11. 37197042 10.4103/ijn.ijn_158_22 PMC10185012

[ref29] Mithra P Ravindra P Unnikrishnan B : Perceptions and attitudes towards organ donation among people seeking healthcare in tertiary care centers of coastal South India. *Indian J. Palliat. Care.* 2013;19(2):83–87. 10.4103/0973-1075.116701 24049347 PMC3775029

[ref30] Mohan L Thanga T Selvam P : Perspective on organ donation in India: a comprehensive review. *J. Community Heal Manag.* 2020;7(3):73–76. 10.18231/j.jchm.2020.017

[ref31] Morgan S Miller J : Communicating about gifts of life: The effect of knowledge, attitudes, and altruism on behavior and behavioral intentions regarding organ donation. *J. Appl. Commun. Res.* 2002;30(2):163–178. 10.1080/00909880216580

[ref32] Meier DE Schulz K Kuhlencordt R : Effects of an educational segment concerning organ donation and transplantation. *Transplant. Proc.* 2000;32:62–63. 10.1016/S0041-1345(99)00879-9 10700971

[ref33] Morgan SE Miller J : Beyond the organ donor card: The effect of knowledge, attitudes, and values on willingness to communicate about organ donation to family members. *Health Commun.* 2001;14:121–134. 10.1207/S15327027HC1401_6 11853207

[ref34] National Organ and Tissue Transplant Organisation: Act and rules of transplant of human organs act (THOA). Reference Source

[ref35] Narendran V Padmavathi S Sangeetha S : Knowledge, awareness and attitude of eye donation among non-clinical staff of a tertiary eye hospital in South India. *Indian J. Ophthalmol.* 2022;70(10):3490–3495. 10.4103/ijo.IJO_725_22 36190032 PMC9789872

[ref37] Patthi B Jain S Singla A : Beliefs and barriers for organ donation and influence of educational intervention on dental students: A questionnaire study. *J. Indian Assoc. Public Health Dent.* 2015;13(1):58. 10.4103/2319-5932.153588

[ref38] Pouraghaei M Tagizadieh M Tagizadieh A : Knowledge and attitude regarding organ donation among relatives of patients referred to the emergency department. *Emergency.* 2015;3(1):33–39. 26512367 PMC4614608

[ref39] Ramadurg UY Gupta A : Impact of an educational intervention on increasing the knowledge and changing the attitude and beliefs towards organ donation among medical students. *J. Clin. Diagn. Res.* 2014;8(5):JC05–JC07. 10.7860/JCDR/2014/6594.4347 24995198 PMC4080019

[ref40] Rithalia A McDaid C Suekarran S : Impact of presumed consent for organ donation on donation rates: a systematic review. *BMJ.* 2009;338:a3162. 10.1136/bmj.a3162 19147479 PMC2628300

[ref41] Sarveswaran G Sakthivel MN Krishnamoorthy Y : Knowledge, attitude, and practice regarding organ donation among adult population of urban Puducherry, South India. *J. Educ. Health Promot.* 2018;7. 10.4103/jehp.jehp_44_18 PMC6149118 30271802

[ref42] Saleem T Ishaque S Habib N : Knowledge, attitudes and practices survey on organ donation among a selected adult population of Pakistan. *BMC Med. Ethics.* 2009;10:1–12. 10.1186/1472-6939-10-5 19534793 PMC2702378

[ref43] Sengul S Sahin MK : The willingness and attitudes of medical students regarding organ donation and transplantation: a cross-sectional study from Turkey. *Rev. Assoc. Med. Bras.* 2022;68:1631–1637. 10.1590/1806-9282.20220307 36449786 PMC9779972

[ref44] Sawhney C Kaur M Lalwani S : Organ retrieval and banking in brain dead trauma patients: Our experience at level-1 trauma centre and current views. *Indian J. Anaesth.* 2013;57(3):241–247. 10.4103/0019-5049.115599 23983281 PMC3748677

[ref45] Spigner C Weaver M Cardenas V : Organ donation and transplantation: ethnic differences in knowledge and opinions among urban high school students. *Ethn. Health.* 2002;7(2):87–101. 10.1080/1355785022000038579 12511196

[ref46] Teoh JYC Lau BSY Far NY : Attitudes, acceptance, and registration in relation to organ donation in Hong Kong: a cross-sectional study. *Hong Kong medical journal = Xianggang yi xue za zhi.* 2020;26(3):192–200. 10.12809/hkmj198176 32448810

[ref47] The Transplantation of the Human Organs Act:1994. ([Last accessed on March 16, 2023). Reference Source

[ref48] Thompson TL Robinson JD Kenny RW : Family conversations about organ donation. *Prog. Transplant.* 2004;14(1):49–55. 10.1177/152692480401400108 15077738

[ref49] Topic I Brkljacic T Grahovac G : Survey of medical students about attitudes toward organ donation. *Dialysis & transplantation.* 2006;35(9):571–574. 10.1002/dat.20046 17639633

[ref50] Tamuli RP Sarmah S Saikia B : Organ donation–“attitude and awareness among undergraduates and postgraduates of North-East India”. *J. Family Med. Prim Care.* 2019;8(1):130. 10.4103/jfmpc.jfmpc_206_18 30911493 PMC6396593

[ref51] Thangavel P Pathak P Chandra B : Millennials and Generation Z: A generational cohort analysis of Indian consumers. *BIJ.* 2021;28:2157–2177. 10.1108/BIJ-01-2020-0050

[ref53] U.S. Department of Health and Human Services, Health Resources and Services Administration, Healthcare Systems Bureau: *National Survey of Organ Donation Attitudes and Behaviors.* Rockville, MD: U.S. Department of Health and Human Services;2013. Reference Source

[ref52] Vijayalakshmi P Sunitha TS Gandhi S : Knowledge, attitude and behaviour of the general population towards organ donation: An Indian perspective. *Natl. Med. J. India.* 2016;29(5):257–261. 28098078

[ref54] Vincent BP Randhawa G Cook E : Barriers towards deceased organ donation among Indians living globally: an integrative systematic review using narrative synthesis. *BMJ Open.* 2022;12(5):e056094. 10.1136/bmjopen-2021-056094 35623762 PMC9150163

[ref55] Wasko MM Faraj S : Why should I share? Examining social capital and knowledge contribution in electronic networks of practice. *MIS Q.* 2005;29:35–57. 10.2307/25148667

[ref56] Watts G : Joseph Murray: innovative surgeon and pioneer of transplantation. *Lancet.* 2011;377(9770):987. 10.1016/S0140-6736(11)60375-9 21420544

[ref57] Wu AMS Tang CS : Solving the dilemma: family communica-tion about organ donation among Chinese, Japanese, and Cau-casian American College students. *J. Appl. Soc. Psychol.* 2009;39(7):1639–1659. (1) (PDF) A Systematic Literature Review and Research Agenda for Organ Donation Decision Communication. [accessed Dec 22 2022]. 10.1111/j.1559-1816.2009.00498.x Reference Source

[ref58] World Health Organization: Human organ and tissue transplantation. 2020. Reference Source

[ref59] World Health Organization: The International report on Organ Donation and Transplantation Activities. 2022. Reference Source

