# Supplementary material for: An empirical investigation on the impact of attitudes towards organ donation in India
Source: F1000Res. 2023 Jun 5;12:463. Originally published 2023 May 3. [Version 2] doi: 10.12688/f1000research.131652.2 (PMC10317467; doi:10.12688/f1000research.131652.2)
Supplement: Peer review history

# Peer review: Reviewer response for version 2

**DOI:** 10.5256/f1000research.148586.r176798

**Published:** 2023-06-15

**Authors:** Alok Atreya

**Affiliations:** 1 Lumbini Medical College, Palpa, Nepal

Dear Authors,

 Thank you for your detailed response to my feedback. I appreciate the efforts you have made to address the concerns and improve the manuscript. Based on your responses, I believe the manuscript has significantly benefitted and is now ready for indexing. 

 I agree with the changes you have made to the introduction section, including the incorporation of the research objective derived from the research questions. This addition helps to clarify the aims and objectives of your study.

 Regarding the sampling technique, I appreciate the clarification provided on the selection of the four malls. The explanation of using footfalls as a metric to identify popular malls and reflect homogeneity in tenant mix and visitors to the mall is appropriate. Additionally, the mention of employing a simple random technique to identify the malls in Dakshina Kannada and Udupi districts for data collection further enhances the transparency and rigour of your methods.

 I am also satisfied with the explanation you provided for the sample size calculation. The use of the proposed approach by Hair, Sarstedt, Ringle, and Gudergan (2017) to determine the sample size based on the number of items on the rating scale multiplied by 10 is appropriate. Your justification for considering 10% of the unanswered sample and resulting in a final sample size of 259 participants is sound.

 I appreciate your efforts in providing additional details on the questionnaire used for data collection. The inclusion of construct-wise details in the Data Gathering section is helpful. Moreover, the fact that the detailed questionnaire is publicly available through Figshare, with the DOI mentioned in the manuscript, ensures transparency and allows for future replication and validation of the instrument. The mention of the reliability and validity of the scales being assessed through the pilot study, with the results presented in the "Data Collection" section, further strengthens the robustness of your methods.

 Regarding the choice of statistical analysis methods, I understand and support your decision to use SmartPLS4 for PLS-SEM analysis, considering the blend of probability and nonprobability sampling techniques and the presence of a mediator in your conceptual framework. The mention of jamovi as an alternative free-source software for structural equation modelling provides valuable information for researchers who may want to replicate or build upon your study using different analytical tools.

 I am glad to see that you have addressed the biases by presenting them as limitations of your research work in the Results section. By acknowledging these limitations, you demonstrate a clear understanding of the potential impact on the generalizability and interpretation of your findings.

 Lastly, I appreciate the improvements made in the discussion section, particularly the mention of cross-sectional research design and the suggestions for future research directions, including experimental and qualitative research. The clarification on the generalizability of the results within the defined scope and research setting, as well as the suggestion for future research from a geographical perspective, adds value to your study.

 In summary, I believe the manuscript has significantly improved based on your responses. You have adequately addressed the concerns raised, providing additional information, clarification, and justifications where needed. Given the changes made, I am pleased to approve the manuscript for indexing.

 Once again, I commend you on your diligent revisions, and I look forward to seeing your research contribute to the field of organ donation in India.

Is the work clearly and accurately presented and does it cite the current literature?

Yes

If applicable, is the statistical analysis and its interpretation appropriate?

Yes

Are all the source data underlying the results available to ensure full reproducibility?

Yes

Is the study design appropriate and is the work technically sound?

Yes

Are the conclusions drawn adequately supported by the results?

Yes

Are sufficient details of methods and analysis provided to allow replication by others?

Yes

Reviewer Expertise:

My area of research is Forensic Medicine & Toxicology. Other broad areas of interest are injury, violence, trauma, abuse, medical ethics, medical education, forensic pathology, forensic anthropology, criminal law, etc.

I confirm that I have read this submission and believe that I have an appropriate level of expertise to confirm that it is of an acceptable scientific standard.

# Peer review: Reviewer response for version 2

**DOI:** 10.5256/f1000research.148586.r176799

**Published:** 2023-06-12

**Authors:** Soumya Swaroop Sahoo, Madhur Verma

**Affiliations:** 1 Department of Community & Family Medicine, All India Institute of Medical Sciences, Bathinda, Punjab, India; 2 All India institute of medical sciences Bathinda, Bathinda, India

I thank the authors for their detailed response to the reviewer's comments. The authors have made the necessary changes in the manuscript in line with the suggestions.

Is the work clearly and accurately presented and does it cite the current literature?

Yes

If applicable, is the statistical analysis and its interpretation appropriate?

Yes

Are all the source data underlying the results available to ensure full reproducibility?

Yes

Is the study design appropriate and is the work technically sound?

Yes

Are the conclusions drawn adequately supported by the results?

Yes

Are sufficient details of methods and analysis provided to allow replication by others?

Yes

Reviewer Expertise:

My areas of research are Non-Communicable Diseases (Cancer, Diabetes), HIV, Tuberculosis, One Heath, and Health systems.

We confirm that we have read this submission and believe that we have an appropriate level of expertise to confirm that it is of an acceptable scientific standard.

# Peer review: Reviewer response for version 1

**DOI:** 10.5256/f1000research.144514.r171950

**Published:** 2023-05-16

**Authors:** Soumya Swaroop Sahoo, Madhur Verma

**Affiliations:** 1 Department of Community & Family Medicine, All India Institute of Medical Sciences, Bathinda, Punjab, India; 2 All India institute of medical sciences Bathinda, Bathinda, India

**Title:**

- The study was conducted in two districts of India, so writing it as attitude towards Organ Donation in India is misleading. The authors can write as " South India"

**Abstract:**

- The abstract is structured and well articulated. However, they need to mention the actual numbers and percentages in the results section rather than " *Awareness of organ donation law in India is low on specific issues and respondents from the health science & medicine discipline scored better on knowledge about organ donation. The findings show that most participants had heard about organ donation and had a favourable attitude toward it."*

**Introduction: **

- The introduction is extensive and aptly describes the background and rationale of the study.

**Methods:**

- The sample size calculation with formula needs justification.
- The post positivism approach needs to be explained more to justify its use here.
- How were the malls selected in the two cities? Justify.
- Since apparently malls have visitors from urban population, it should be mentioned in the limitations.

**Results:**

- The results are well described with the appropriate use of statistical tests.
- *"A “chi-square test of independence” displayed that there was a significant association between education and awareness of the OTD act in India Χ2 (1, N = 259) = 6.980, p = 0.06)."* - How was this significant as a p-value less than 0.05 is considered significant?

**Discussion:**

- The discussion might include more studies conducted on the Indian population in recent times.
- Biases affecting the study results should be mentioned in the limitations.

**Overall:**

 The manuscript is well written with a comprehensive literature review, good statistical analysis, and clear interpretation of the results. However, the gaps and suggestions outlined above might be given consideration.

Is the work clearly and accurately presented and does it cite the current literature?

Yes

If applicable, is the statistical analysis and its interpretation appropriate?

Yes

Are all the source data underlying the results available to ensure full reproducibility?

Yes

Is the study design appropriate and is the work technically sound?

Yes

Are the conclusions drawn adequately supported by the results?

Yes

Are sufficient details of methods and analysis provided to allow replication by others?

Yes

Reviewer Expertise:

My areas of research are Non-Communicable Diseases (Cancer, Diabetes), HIV, Tuberculosis, One Heath, and Health systems.

We confirm that we have read this submission and believe that we have an appropriate level of expertise to confirm that it is of an acceptable scientific standard, however we have significant reservations, as outlined above.

# Response

**Published:** 2023-05-22

**Authors:** smitha nayak

**Affiliations:** Manipal University, India

We thank you for your valuable feedback. We have discussed the feedback and incorporated changes, wherever necessary, in the manuscript. We present our response to your comments below:

 1. Abstract: The quantified output is presented in the abstract and changes are incorporated in the manuscript. This has added value and brought clarity to the manuscript.

 Methods :

 2. Sample size calculation. The sample size was calculated based on the number of items on the rating scale multiplied by 10 as proposed by Hair, Sarstedt,Ringle, & Gudergan(2017). The same has been incorporated in the manuscript and added to the reference list.(Hair JF Jr, Sarstedt M, Ringle CM, et al.: Advanced issues in partial least squares structural equation modeling. Sage Publications; 2017)

 3. Post positivism approach: The philosophical assumption in the backdrop of Post positivism approach is that consent from the participants has been taken to uphold the nature of research ethics and it is primarily quantitative nature of inquiry. Hence, we have undertaken this research in Post positivism approach. The same has been incorporated in the manuscript.

 4. Malls selection: Footfalls is a metric that indicates the popularity of the mall and also reflects homogeneity in tenant mix and visitors to the mall (Mohan and Tandon, 2015). Hence 4 malls in each district with the highest footfalls were identified to undertake the survey. Further, a simple random technique was employed to identify the malls for data collection.

 Mohan, M., & Tandon, K. (2015). Mall Management: An analysis of customer footfall patterns in Shopping Malls in India.  *International Journal of Scientific and Research Publications*, *5*(3), 1-15.

 5. Limitations: Since apparently malls have visitors from the urban population, it should be mentioned in the limitations.

 This has been addressed and mentioned in the limitations section of the manuscript.

 6. Results:

 The results are well described with the appropriate use of statistical tests.

  "A “chi-square test of independence” displayed that there was a significant association between education and awareness of the OTD act in India Χ2 (1, N = 259) = 6.980, p = 0.06)." - How was this significant as a p-value less than 0.05 is considered significant?

 Due apologies for the typo error. We have incorporated the changes.

 7. Overall Structure :

 The purpose of the research is strengthened in the manuscript.

 We hope we have incorporated/justified the revisions made in the manuscript.

# Peer review: Reviewer response for version 1

**DOI:** 10.5256/f1000research.144514.r171952

**Published:** 2023-05-12

**Authors:** Alok Atreya

**Affiliations:** 1 Lumbini Medical College, Palpa, Nepal

**Abstract:**

 The abstract is well-structured, presenting the background, methods, and results. It provides a clear overview of the study's objectives and findings.

**Introduction:**

 The manuscript provides a clear and detailed introduction to the topic of organ donation and transplantation, including the current state of affairs, challenges faced, and existing initiatives undertaken by governments and NGOs to promote organ donation.

**Methodology:**

 The methodology section is also comprehensive, describing the study design, data gathering, and data analysis techniques used. However, there are some areas where the manuscript could benefit from improvement or clarification.

1. The manuscript would benefit from a clear and concise research question, which is missing from the introduction section. While the overall goal of the study is clear, a specific research question would help to focus and guide the study and make the authors' aims and objectives more explicit.
2. The sampling technique used could be improved. The authors state that a purposive sampling approach was adopted due to the absence of a sampling frame, but it is unclear how the four shopping malls were selected. It would have been better to state clearly the criteria for selecting these malls and why they were chosen. Additionally, the sample size calculation could be explained more clearly and justified in the methods section.
3. The authors could provide more detail on the questionnaire used for data collection. While the items included in the questionnaire are briefly described in the methods section, a clearer explanation of how the questionnaire was developed and validated would help to establish its reliability and validity.
4. The authors should provide more detail on the data analysis methods used. While it is stated that SmartPLS4 and jamovi were used for data analysis, the statistical tests used, and their justification should be described in more detail.

**Results:**

 This section is well-written and provides a comprehensive analysis of the research study findings. The authors use appropriate statistical methods to analyze the data and report their results in a clear and concise manner. However, it is essential to note that the manuscript section does not provide information on the sampling strategy used to select the participants, which can impact the generalizability of the results. Additionally, the authors do not mention any potential biases that may have affected the results, such as response bias or social desirability bias.

**Discussion:**

 There are also some limitations to the study that need to be considered. The cross-sectional research design can limit the ability to establish causal relationships or determine changes over time. Moreover, the study participants were only from urban areas, so the findings may not be generalizable to the entire population. Lastly, the manuscript did not provide detailed information on the sample size or the sampling procedure, which makes it difficult to evaluate the representativeness of the sample or the potential for bias.

**In General:**

 The manuscript has several strengths, including a clear and concise writing style, a comprehensive literature review, and a well-organized presentation of the study results. The authors also make some important recommendations for future research, including exploring the knowledge, attitudes, and intentions of organ donation across different generations and investigating the reasons for the low donation rates in India.

 Overall, the manuscript is well-written and provides valuable insights into the knowledge, attitudes, and intentions of organ donation in India. However, the limitations of the study should be considered, and future research should address these limitations to build on the findings presented in this manuscript.

Is the work clearly and accurately presented and does it cite the current literature?

Yes

If applicable, is the statistical analysis and its interpretation appropriate?

Yes

Are all the source data underlying the results available to ensure full reproducibility?

Yes

Is the study design appropriate and is the work technically sound?

Yes

Are the conclusions drawn adequately supported by the results?

Yes

Are sufficient details of methods and analysis provided to allow replication by others?

Yes

Reviewer Expertise:

My area of research is Forensic Medicine & Toxicology. Other broad areas of interest are injury, violence, trauma, abuse, medical ethics, medical education, forensic pathology, forensic anthropology, criminal law, etc.

I confirm that I have read this submission and believe that I have an appropriate level of expertise to confirm that it is of an acceptable scientific standard, however I have significant reservations, as outlined above.

# Response

**Published:** 2023-06-14

**Authors:** smitha nayak

**Affiliations:** Manipal University, India

Thank you for the feedback provided for our manuscript; each has significantly added value to it. We have responded to each one of them and presented our responses below:

 1. Introduction: no changes suggested 

 2. Methodology: 

 a. The research objective derived from the research questions has been incorporated in Pg 4 of the manuscript. The need for the study has been included as follows: "Hence it is imperative to assess the current knowledge level, attitude, and willingness to register for organ donation among various age groups and whether it varies across the population with the discipline of education"

 b. Sampling: Clarity on each point and the appropriate changes incorporated in the manuscript

 Mall identified: " Footfalls are a metric that indicates the popularity of the mall and also reflects homogeneity in tenant mix and visitors to the mall ( Mohan and Tandon, 2015). Hence 4 malls in each of the district s having the highest footfalls were identified to undertake the survey. Further , a simple random technique was employed to identify the malls in Dakshina Kannada and Udupi districts for data collection

 c: Sample size calculation: " The sample size was calculated based on the number of items on the rating scale multiplied by 10 as proposed by Hair, Sarstedt, Ringle, & Gudergan(2017). Hence, The sample size was determined by multiplying the number of rating scale elements by ten [15] i.e. 15*10 = 150. Considering 10% of the unanswered sample (i.e.15) gives rise to 165 (150 + 15 = 165). Finally, 259 participant data were collected."

 3: Questionnaire: Construct wise details have been presented in the Data Gathering section of the manuscript. The items of each construct ( detailed questionnaire) is uploaded in Figshare, hence publicly available. The DOI of the same has been mentioned in the manuscript. 

 The reliability and validity of the scales was done through the pilot study, the results of which are presented in the "Data Collection" section of the manuscript.

 4 PLS: as we have opted for a blend of probability and nonprobability sampling techniques, and the presence of a mediator in the conceptual framework, PLS-SEM was the tool chosen against Jamovi or AMOS. 

 "To test the hypotheses and perform the mediation analysis, the statistical package SmartPLS4 (RRID: SCR 022040) was used. PLS-SEM is becoming more popular as a statistical package due to its versatility and dependability in analyzing composite and empirical studies. Structural Equation Modelling performed in this study can also be undertaken by using jamovi (RRID:SCR_016142), which is a free source software."

 5. Results: The biases are presented as limitations of the research work undertaken. 

 6. Discussion: Cross section research design: has been employed in the study. Experimental and Qualitative research has been suggested as a direction for future research. The results are generalizable as the scope and research setting are justified in the manuscript. Future research is suggested from a geographical perspective of the study.
